# Transforming Mental Health Care – Policy Implementation

**DOI:** 10.1192/j.eurpsy.2025.698

**Published:** 2025-08-26

**Authors:** H. Prata Ribeiro, P. Mackay, E. Azevedo, N. Bettencourt

**Affiliations:** 1 Saúde Mental, Estrutura para a Saúde Mental dos Açores, Angra do Heroísmo; 2 Serviço de Psiquiatria, Hospital Beatriz Ângelo, Loures; 3 Medicina, Católica Medical School, Lisboa, Portugal

## Abstract

**Introduction:**

The Autonomous Region of the Azores faced considerable challenges in providing comprehensive, efficient, and integrated mental health care services, exacerbated by geographical isolation, resource constraints, and the absence of a cohesive system for referrals and quality assessment in mental health care.

**Objectives:**

Despite the recognized need, the integration of mental health services into primary health care (PHC) and the broader health system in the Azores was fragmented. Key issues included lengthy waiting lists, inadequate referral systems between primary care and specialized psychiatric services, and a lack of standardized quality assessment and performance indicators for mental health care. Additionally, there was a significant need for community mental health teams, emergency management of agitated patients, and comprehensive training for health professionals in psychiatric care.

**Methods:**

Through collaborative efforts, the region achieved notable advancements, including the creation of referral criteria from PHC to psychiatry services, structuring inter-service referrals, reactivation of Community Mental Health Teams, and establishment of a “Physician’s Bank.” Noteworthy was the elimination of a waiting list of over 1,000 patients and the development of quality assessment and performance indicators. The construction of a courtyard for involuntarily hospitalized patients and the creation of an emergency room on Faial Island further exemplified the tangible improvements in patient care and system efficiency. Training programs were extensively implemented across various professional groups, enhancing the capacity for mental health care delivery at all levels.

**Results:**

The integration efforts underscored the value of cross-sector collaboration, stakeholder engagement, and the adaptation of models like the SURE framework and COM-B theory to address the multifaceted barriers to mental health care integration. Key facilitators included the development of guidelines, training, and clinical supervision, alongside innovative approaches to public health interventions.

**Conclusions:**

The transformative work in the Azores exemplifies how integrated care models, supported by strategic collaborations and policy reforms, can significantly enhance mental health service delivery in geographically isolated regions. It underscores the importance of systemic approaches to training, infrastructure development, and stakeholder engagement in achieving sustainable improvements in mental health care.

**Disclosure of Interest:**

None Declared

